# Molecular characterization and validation of sunflower (*Helianthus annuus* L.) hybrids through SSR markers

**DOI:** 10.1371/journal.pone.0267383

**Published:** 2022-05-19

**Authors:** Hafiz Ghulam Muhu-Din Ahmed, Muhammad Rizwan, Muhammad Naeem, Muhammad Ahsan Khan, Faheem Shehzad Baloch, Sangmi Sun, Gyuhwa Chung

**Affiliations:** 1 Department of Plant Breeding & Genetics, Faculty of Agriculture and Environment, The Islamia University of Bahawalpur, Bahawalpur, Pakistan; 2 Agricultural Biotechnology Division, National Institute for Biotechnology and Genetic Engineering (NIBGE), Faisalabad, Pakistan; 3 Department of Plant Breeding & Genetics, University of Agriculture, Faisalabad, Pakistan; 4 Faculty of Agricultural Sciences and Technologies, Sivas University of Science and Technology, Sivas, Turkey; 5 Department of Biotechnology, Chonnam National University, Chonnam, Republic of Korea; Government College University Faisalabad, PAKISTAN

## Abstract

Genetic purity is a prerequisite for exploiting the potential of hybrids in cross-pollinated crops, such as sunflower. In this regard DNA-based study was conducted using 110 simple sequence repeat (SSR) markers to check the genetic purity of 23 parents and their 60 hybrids in sunflower. The polymorphism was shown in 92 markers with value 83.63%. The SSR markers ORS-453 and CO-306 showed the highest PIC values of 0.76 and 0.74, respectively. The primer ORS-453 amplified allele size of 310 base pairs (bp) for female parent L6 and 320 bp for L11, while for male parents, T1 and T2 had allele size 350 bp and 340 bp, respectively. The hybrids from these parents showed a similar size of alleles with parents, including hybrids L6×T1 (310 bp and 350 bp), L6×T2 (310 bp and 340 bp), and L11×T2 (320 bp and 340 bp). Similarly, the primer CO-306 amplified allele size 350 bp and 330 bp for female parents L6 and L11, respectively, while, allele size 300 bp and 310 bp for male parents T1 and T2, respectively. The hybrids’ allele size was like the parents *viz*., L6×T1 (350 bp and 300 bp), L6×T2 (350 bp and 310 bp), and L11×T2 (330 bp and 310 bp). All 60 hybrids and their 23 parents were grouped into three main clusters (A, B and C) based upon DARWIN v.6.0 and STRUCTURE v.2.3 Bayesian analyses using genotypic data. Further, each main cluster was divided into two sub-divisions. Each sub-division showed the relatedness of parents and their hybrids, thus authenticating the genetic purity of hybrids. In conclusion, this study provides useful for accurate and effective identification of hybrids, which will help to improve seed genetic purity testing globally.

## Introduction

Sunflower (*Helianthus annuus* L.) is the fifth most important crop among oilseeds and second in hybrid seeds after maize globally. Local production of edible oil in Pakistan during 2020–21 is provisionally estimated at 0.374 million tonnes. Total availability of edible oil from all sources is estimated at 3.291 million tonnes. Sunflower ranks third after cottonseed and rapeseed in Islamic Republic of Pakistan, with a total area of 151 thousand hectares and seed production of 87 thousand tons. Pakistan is far behind in the average yield (1.29 metric tons/ha) compared to other countries, with China leading (2.67 metric tons/ha) followed by Ukraine 2.22 metric tons (Economic Survey of Pakistan 2020–21) [1].

The genetic diversity of cultivated and wild germplasm is characterized for use with various breeding purposes. Molecular markers have been developed to facilitate sunflower breeding. Markers linked to monogenic traits in a population mapping can be used to select plants with those traits. Identified linked markers need to be validated in various genetic backgrounds and environments to ensure widespread utility [2–4]. Genomic sequence data is now accessible for sunflower, which must now be oppressed to grow new SNP based markers associated with genes of interest to mine allelic diversity linked to economically significant characters, specifically characters well deliberate in other organisms, such as seeds oil contents and resistant gene [2, 5]. The development of early maturing and high yielding hybrids are the major objective of sunflower-breeding programs that have been costly and time-consuming. The parental selection with the potential of high yielding hybrids and their purity testing are the constraints in heterosis breeding. The advances in molecular techniques have facilitated this process. Genotyping explores ways for elite germplasm protection and passportisation [6] successful use of genetic distance in hybrid performance prediction and parental selection [7] and identification of valuable traits in the genotypes using marker-assisted selection (MAS) techniques.

Sunflower breeding program has been developed using molecular marker. These marker are highly polymorphic, usually co-dominant, have a strong link with the trait of interest, measurable at all growth stages, and phenotypically neutral [8]. Numerous studies in the sunflower have documented the importance of marker assisted selection (MAS), the estimation of genetic diversity, the identification of integrated lines in the reproduction of heterosis and in the determining of heterotic patterns [9]. Markers potentially suitable for MAS have been identify via Quantitative Traits Loci (QTL) mapping of economically importance trait [9–11]. Commercial production of hybrid seeds depends on Cytoplasmic Male Sterility (CMS) and Male Fertility Restoration (RF) lines [12, 13].

The information about genetic diversity and hybrid identification in the available germplasm and among elite breeding material is essential in plant breeding [14–16]. PCR-based simple-sequence repeat (SSR) markers are co-dominant markers and extensively used in hybrid identification (Chalmers et al., 2001), and the identification of markers is linked to different traits. SSR methodology has been used for pure hybrid analyses in several crops [15].

Different kinds of molecular markers are used in fingerprinting of sunflowers such as AFLP, RAPD, SNPs [17] and SSRs are one of the most frequently used molecular markers for fingerprinting, genome mapping, phylogenetic and population studies, estimation of genetic polymorphism and marker-assisted selection due to simplicity, co-dominant inheritance, reproducibility, high polymorphism and low cost [18, 19]. These characteristics of the SSR markers promoted for various species like barley [20], maize [21], wheat [22], rice [23] rapeseed [24] and others. More possibilities and areas were opened due to the construction of the first SSR genetic map and its use in sunflower experiments such as fingerprinting was broadened.

The traditional Grow out Test (GOT) is done to determine the seed genetic purity test based on morphological markers are time consuming and are environ-mental dependence [25, 26]. To overcome this disadvantage, the biochemical markers are being used in many crops. However, repeatability and accuracy of these results on biochemical markers are subject to question. This made a way for the use of DNA molecular markers particularly the co-dominant markers. The SSR markers are of great importance for rapid assessment of hybrid and parental line seed purity [26, 27].

Therefore, SSR markers have become the most suitable choice for selecting parents and authentication of their hybrids. Thus, this study was conducted with the aim to validate the parents and their hybrids in breeding programs and the genetic distances (clusters) were also used to authenticate the origin of hybrids with their related parents.

## Materials and methods

### Plant material

This research was conducted using 83 genotypes of sunflower containing three fertility restorers (Rf) lines, twenty cytoplasmic male sterile (CMS) lines and their 60 hybrids. The detail information about genotype code, name and their origin record were revealed in S1 Table in S1 File. The crossing plan was according to Line × Tester devised by Kempthorn [28]. The crosses were made in such a way that each male parent was crossed to all female parental lines. The females were assigned to each male parent. Total 60 crosses were made using this design and their phenotypic performance in field were evaluated in another experiment [29] and listed in S2 Table in S1 File. In this study only, validation of Sunflower hybrids through SSR Markers were performed. The plant material was germinated in the culture of half MS (Murashig and Skoog) for ten days under optimum conditions [30].

### DNA extraction

From the lyophilized leaves of ten days old seedling germinated etiolated in the culture medium, genomic DNA was extracted using CTAB standard protocol with few amendments [31]. The quality and quantity of extracted DNA were determined using 3% agarose gel electrophoresis in a TAE system with known concentrated DNA and a Nanodrop ND-1000 spectrophotometer, respectively. Genomic DNA of all the 83 (23 Parents and 60 Crosses) genotypes visualized on gel electrophoresis as mentioned in S1 Fig in S1 Raw images. For further analysis, DNA was used in Polymerase Chain Reaction (PCR).

### PCR conditions

The reaction mixture of 10 μl containing 1x PCR buffer with (NH_4_)_2_SO_4_, 50–100 ng of DNA, 2 mmol/L MgCl_2_, 0.6 μmol/l of each primer, 1μl of 0.25 mmol/L dNTPs and 1.25 μl *Taq* DNA polymerase in MyCycler^TM^ Thermal cycler (Bio-Rad). The conditions used were 94°C for 120 sec, 1 cycle was run at 94°C for 30 sec, 63°C for 30 sec and 72°C for 45 sec, 5 cycles were run at the conditions as 94°C for 30 sec, 62°C for 30 sec (−1°C/cycle) and 45 sec at 72°C which was followed by 30 cycles of the conditions as 94°C for 30 sec, 57°C for 30 sec and 72°C for 45 sec and final extension phase for 300 sec at 72°C. The primer names and their sequences of polymorphic markers are listed in S3 Table in S1 File. The primers were selected from scholarly publications and the Sunflower Database (http://sunflower.uga.edu/cmap/) depending upon their successful application in sunflower genotyping and their level of polymorphism.

### SSR data analysis

The results obtained through amplicons’ analysis were scored with 1 [presence] and 0 [absence] of these alleles and further aligned for genetic characterization. The total number of alleles per locus and allele frequency was calculated by the statistical analysis software GenAlEx version 6.5. The software version 3.23 of POWER MARKER was used for PIC (Polymorphic Information Content) values and gene diversity calculation. The UPGMA (unweighted pair group method with arithmetic mean) method used for cluster analysis running DARWIN software version 6.0. To identify the clusters of similar individuals, the Bayesian clustering method was applied using STRUCTURE version 2.3 statistical software [32]. In the STRUCTURE program, a burn-in length of 10^4^ cycles to minimize the starting configuration effect, 10^6^ cycles of simulation run, and the admixture model option applied. STRUCTURE HARVESTER version 0.6.93, a web-based software, was used to derive the optimal number of clusters or peaks “K” This allows the STRUCTURE-based results to visualize and understand the cluster numbers on ad-hoc based techniques [33]. We chose the cluster values (K) in the range of 1–10 with six independent runs for each value to get consistent results.

## Results

To study genetic diversity and variability among plant populations of similar species or different plant species, molecular markers are considered to be a versatile tool. Conventionally, the best parents are selected based on the combining ability analysis for hybridization. However, this procedure is very time consuming and less reliable because of higher environmental influences. The alternative tool which is more efficient, and time-saving is the utilization of the molecular markers for the identification of better yielding hybrids through selection and hybridization of potential inbred lines. The microsatellites (SSR) give a better way to be utilized for exploring variation in parental lines of breeding material on the basis of multiallelic and polymorphic nature of these markers. The major advantage over conventional methods for using DNA markers lies in the stability due to being unaffected by environmental effects.

**Fig 1:**
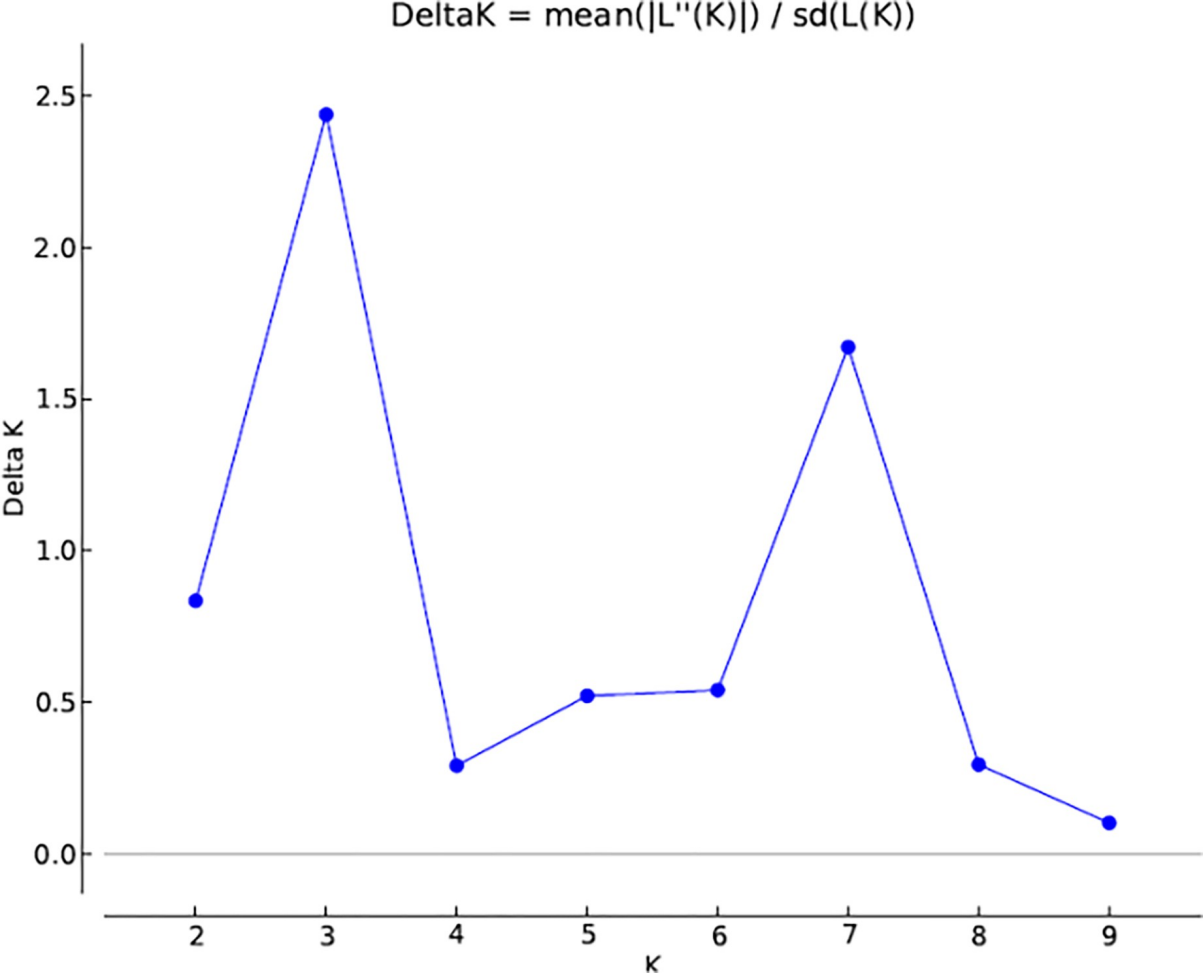
The result obtained of 83 genotypes (23 parents and 60 hybrids) using 92 SSR molecular markers using Structure Harvester analysis.

**Fig 2:**
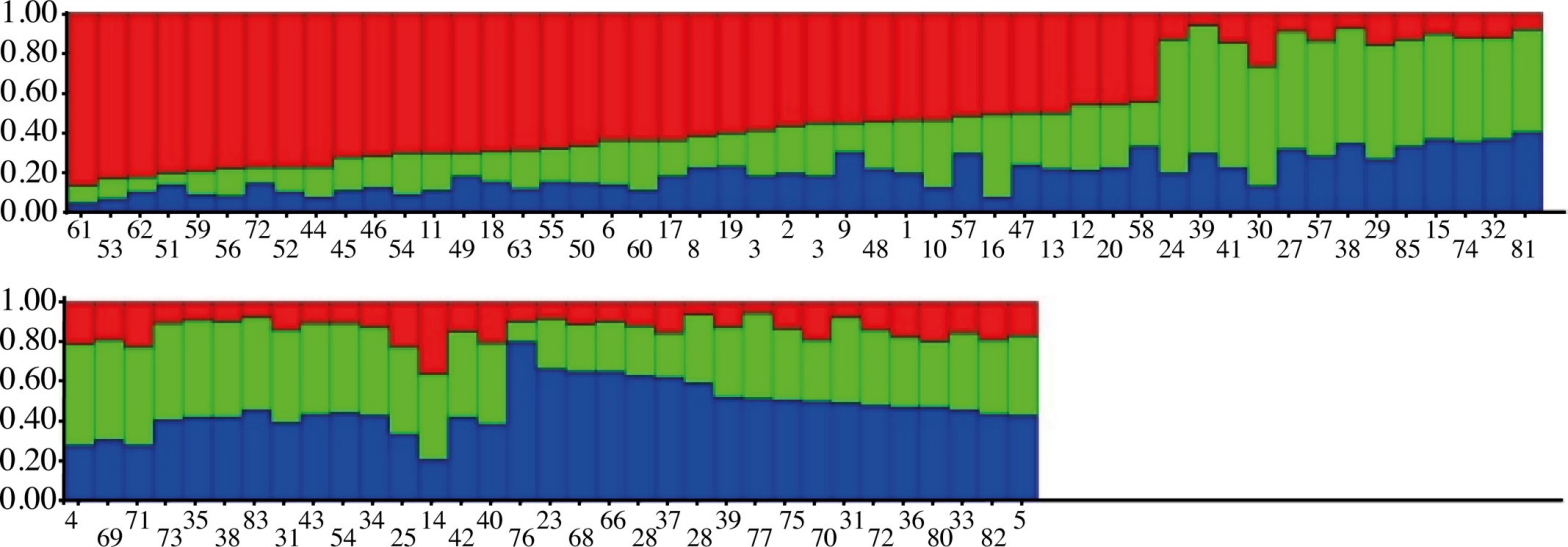
Genetic relatedness of individuals from 83 sunflower populations using Structure software. Numbers on y-axis show the membership coefficient to sub-populations and numbers on x-axis show the individual code belongs to sunflower populations.

The genetic divergence among parental lines was also estimated in this study using simple sequence repeat (SSR) markers. The sunflower populations were divided into three subpopulations based upon Structure analysis. Bayesian method in the Structure software was used for effective and efficient analysis of the whole population structure which classified all the individuals into appropriate subpopulations. This method of clustering is based upon the method of allocation of individual genotypes to K clusters in such a manner that within clusters, Hardy-Weinberg law of equilibrium and linkage equilibrium are valid while absent between clusters. In this study, the maximum value of K was observed in K = 3, thus subdividing the studied population into three subpopulations (Fig 1). Plant populations’ genetic structure is indicative of interactions for various factors such as individual’s genetics, the long history about the evolutionary studies species and migration of genes within and between species. Genetic relatedness of individuals from 83 sunflower populations using Structure software. Numbers on y-axis show the membership coefficient to sub-populations and numbers on x-axis show the individual code belongs to sunflower populations Fig 2.

Among 110 SSR markers used, 92 were found dominant in parents, which showed single allele and co-dominant in hybrids showing both alleles from related parents. The molecular marker ORS-453 gave amplicon size of alleles 310 base pairs (bp) for line L6 (female parent) and 320 bp for L11 female parent while for male parents, T1 (tester1) and T2 (tester2) had allele sizes 350 bp and 340 bp, respectively. The developed hybrids from these parents showed the same size of alleles as the parents, like hybrids L6×T1 (310 bp and 350 bp), L6×T2 (310 bp and 340 bp), and L11×T2 (320 bp and 340 bp). Similarly, the primer C0306 gave amplification of allele size 350 bp and 330 bp for female parents L6 and L11, respectively, while, allele size 300 bp and 310 bp for male parents T1 and T2 respectively *viz*., L6×T1 (350 bp and 300 bp), L6×T2 (350 bp and 310 bp), and L11×T2 (330 bp and 310 bp).

Out of 110 SSR markers used in this study for genotyping of sunflower hybrids and their parents, 92 markers showed high polymorphisms that indicated the variations in studied germplasm. These polymorphic markers were 83.63% of total markers. The highest PIC value was 0.76 with primer pair ORS-453 and the lowest value was 0.64 with ORS-1040 with mean value 0.69 (S4 Table in S1 File). The remaining 18 molecular markers showed monomorphism, which were non-significant for molecular analysis in this experiment. A total number of alleles detected were 405 with an average number of 4.40 alleles per marker. These markers, in combination and individually, created bands of a specific pattern for each specific hybrid and its parents. Thus, this study revealed 92 markers to be suitable for DNA fingerprinting for hybrid validation in sunflower. SSR markers are useful for a variety of applications in plant breeding and genetics because of their reproducibility, multiallelic nature, codominant inheritance, relative abundance, and good genome coverage. These markers are an efficient tool for hybrid authentication.

**Fig 3:**
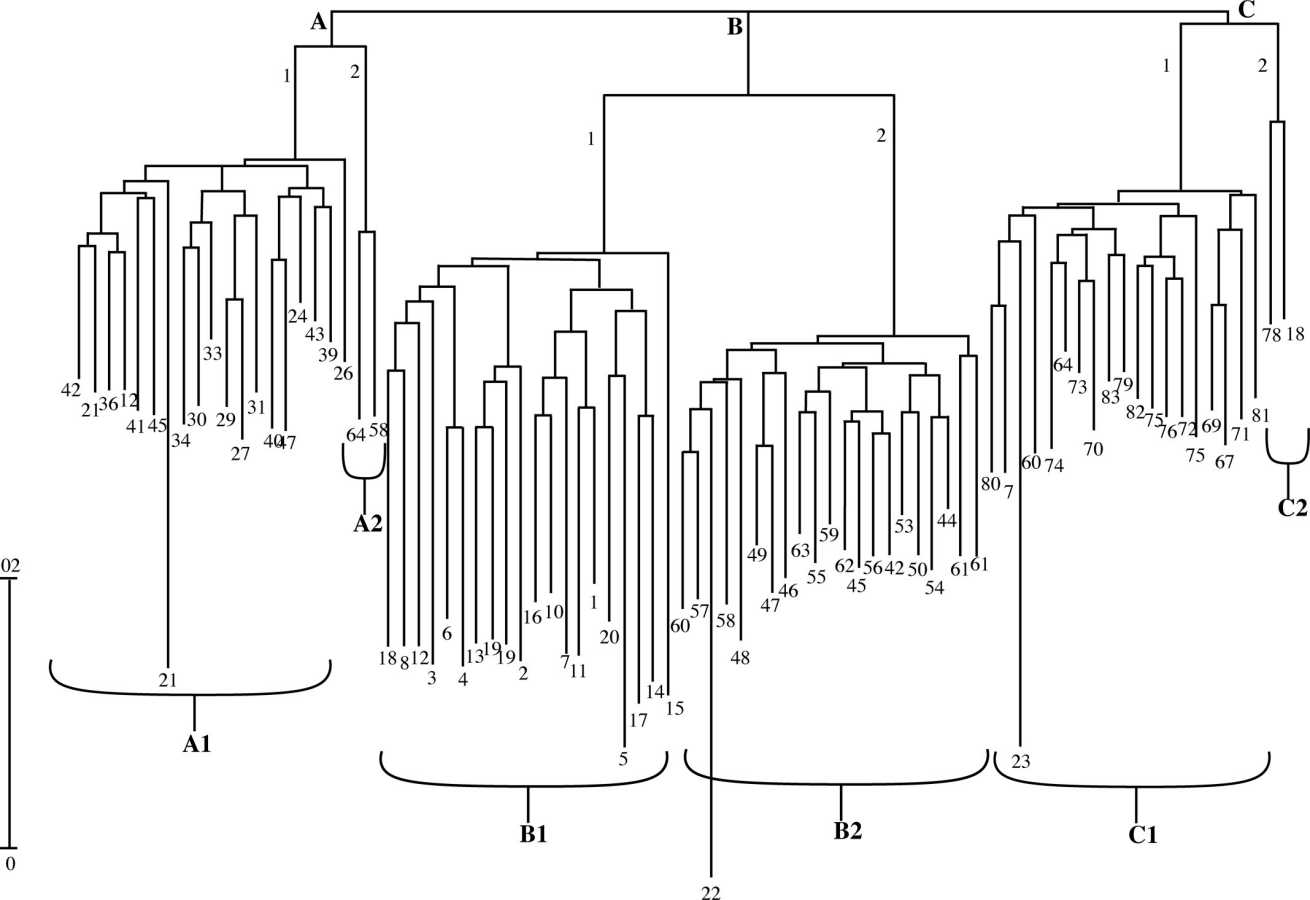
Dendrogram of sunflower genotype distribution based on microsatellite markers.

In the currently analyzed sunflower genotypes, the SSR analysis showed both heterozygous and homozygous states of the loci investigated. To understand this, we take the example of the primer pair ORS-453 in this study, which generated 6 alleles of 300–350 bp (heterozygous state of the locus) in the studied genotypes (S2 Fig in S1 Raw images). Of all markers used in the study, 92 were polymorphic and exhibited useful results, thus considered fit for fingerprinting in the heterozygous state, possibly due to the heterogeneity of the investigated genotypes and duplications of microsatellite loci or maybe it defines the genetic impurity of selected materials. Cluster analysis revealed the genetic distance between parents and their hybrids (Fig 3).

The dendrogram results show that the paternal and maternal genotypes, as well as their hybrids, were clearly distinguished (Fig 3). Overall, three main clusters (A, B & C) were identified, the first one (A1) showing 18 hybrids from female parents (lines) crossed with male parent T1 (tester1) designated as number 21 in A1 dendrogram); the second one (B) was divided into two sub-clusters 1&2, where sub cluster1 consisted of all the female lines named as B1 and sub-cluster 2 consisted of all the hybrids named as B2 with the male parent T2 (tester2) at position 22 in B2 dendrogram; and third one (C) consisted of two sub-clusters, C1 containing the hybrids and their male parent T3 (tester3) at position 23 in C1 sub-cluster except for sub-cluster C2 in which the female line numbered as 18 and hybrid numbered as 78 were grouped similarly due to various genetic constitutions from other groups only based upon these 92 polymorphic markers. The presence of testers in different clusters also indicates their broad genetic base, which is a requirement for the testing of female parents in breeding programs.

The results based on SSR molecular analysis clearly reveal that the ability of SSRs, used in this study to authenticate the hybrids, can also be validated in varietal discrimination, seed genetic purity testing and DUS (Distinctiveness, Uniformity, and Stability) studies in varietal development procedure. However, the SSR markers require identification of hetero-zygosity of the sunflower hybrids and distinguishing the hybrid in question from its parental lines and other hybrids. Therefore, the present study was conducted to identify a specific SSR marker to discriminate the sunflower hybrids form its parental lines.

## Discussion

Molecular markers are crucial for understanding genome organization and provide important advantages in the means of development of new lines and determination of differentiation between initial germplasm [34, 35]. The development of molecular markers in sunflower is at an advanced level and different types of markers have been developed for marker-assisted selection (MAS) over the years [7]. There are numerous different molecular markers available which can be used in sunflower breeding [36]. In current experiment, DNA of 23 sunflower parents and their 60 hybrids was amplified with 92 different polymorphic SSR markers (Table 1). The number and size of the DNA fragments were strictly dependent on the sequence of the primer. Reactions were repeated two to three times to check the consistency of the amplified products, and only easily resolved and bright DNA bands were counted. All F_1_ genotypes showed an authentic confirmation with their parents in current study. These results suggest that SSR markers provide information for the identification of sunflower parents and their hybrids [9]. Today, conventional methods of purity testing such as grow out turn (GOT) are not encouraging because of their time-consuming factors and environmental influence on morphological characters. The alternative approach is the application of molecular markers for varietal identification, hybrid confirmation and parental selection for hybridization programs. Genetic impurity of hybrids is a widely spread issue in cross-pollinated crops like sunflower while morphological description and discrimination based merely upon phenotype, are clumsy and less reliable [37].

**Table 1:** Scorable DNA bands amplified by polymorphic SSR primers through PCR.

Marker name	ORS453	CO306
**BP**	300–350	350–390
**PIC**	0.76	0.7
**GD**	0.72	0.72
**TNA**	6	5
**Forward Primer**	CCTGTGAGCTACAATACTCCCACA	ACTGTCAACACCTCCTTCGACT
**Reverse Primer**	GATTCTGATTAGGCGGTGGT	GGCTTACACTTCTCTCCATCTCAA

BP = Base pair position, PIC = Polymorphic Information contents, GD = Gene Diversity, TA = Total number of Alleles

Genetic diversity and population have differences in them. Structure and other such methods are based upon population genetics model which is interpretable while for genetic diversity of primitive evolution, Darwin was used [38]. In this study, all 60 hybrids and their 23 parents were grouped into three main clusters (A, B and C) based upon DARWIN v.6.0 and STRUCTURE v.2.3 as shown in Fig 2. Bayesian analyses using genotypic data. Further, each main cluster was divided into two sub-divisions. Each sub-division showed the relatedness of parents and their hybrids, thus authenticating the genetic purity of hybrids. The identification of subgroups which are highly consistent could be facilitated by comparison of accessions distribution by both analyses as Structure software and Neighbor-Joining tree (Darwin software). To conserve germplasm of plant resources, the genetic distance between plant populations can be a valuable and important parameter. It was proven that crosses formed by genetically distant and unrelated parents show better and more powerful hybrids as compared to the crosses or hybrids of closely related genotypes [2] Thus, the genetic analysis of population structure separated the population into breeding subpopulations.

To fully exploit the genetic potential of heterosis, it is necessary to characterize the parental material at morphological and genetic levels. The study of genetic divergence helps breeder focus some possible promising lines for hybrid breeding. In the current experiment, DNA molecular marker system of SSRs were used for genetic relationships and hybrid identification with their respective parents in sunflower. The total number of alleles per locus (6.67) was observed highest compared to 2.32 alleles and 3.5 alleles per locus by, respectively. A high number of alleles per locus could be a result of natural outcrossing as the sunflower is cross-pollinated crop who observed 5.57 alleles per locus. All the markers are not useful for genotyping, varietal identification and genetic distance. The markers’ strength can primarily be determined by its PIC value, greater the PIC value, stronger will be a marker for genotypic discrimination of sunflower. In current study PIC values revealed a significant positive association with the gene diversity (GD) and total numbers of allele for SSR markers. All the results (GD, TNA and PIC values) suggested the existence of genetic diversity among parents. The allelic SRBP (size range in base pairs) also strongly associated with the TNA which is closed to the previously reported findings [7, 27]. The results reported by wheat scientists about strong association of TNA and allelic SRBP also supported the results of current study [39].

The most powerful marker was ORS-453 with 0.76 PIC value whereas, the weakest marker with the lowest PIC value 0.64 was ORS-1040 in the current studies. The markers showing low PIC values must be avoided in genomic studies especially less than 0.2 The average PIC value found in this study was 0.59, The genetic distances shown by dendrogram reported the clusters of the hybrids along with their male parents (testers) in separate clusters (Fig 3). In the parental lines, there must be adequate genetic information to select for crossing the material that would accelerate hybrid vigor. Among 110 SSR markers used, 92 were found dominant in parents, which showed single allele and co-dominant in hybrids showing both the alleles from related parents.

The molecular marker ORS-453 gave amplicon size of alleles 310 base pairs (bp) for line L6 (female parent) and 320 bp for L11 female parent while for male parents, T1 (tester1) and T2(tester2) had allele sizes 350 bp and 340 bp, respectively. The developed hybrids from these parents showed the same size of alleles as the parents, like hybrids L6×T1 (310 bp and 350 bp), L6×T2 (310 bp and 340 bp), and L11×T2 (320 bp and 340 bp). Similarly, the primer C0306 gave amplification of allele size 350 bp and 330 bp for female parents L6 and L11, respectively, while, allele size 300 bp and 310 bp for male parents T1 and T2 respectively *viz*., L6×T1 (350 bp and 300 bp), L6×T2 (350 bp and 310 bp), and L11×T2 (330 bp and 310 bp). (S1 Fig in S1 Raw images). 250–300 and 200–300 size (S1 Fig in S1 Raw images). Reported amplicon size of 250–300 bp and 200–300 bp for hybrid authentication using SSR markers. Previously scientists [40] confirmed the hybrid (*H*. *rigidus × H*. *annuus*) with their related species by using SSR markers. Previously scientists [6] reported that SSR makers gave amplicon sizes of 114 bp and 156 bp in their study of sunflower fingerprinting. So, molecular markers are a good tool for hybrid authentication.

In current experiment, distances among cluster or group clearly showed the variations between male and female parents and all subgroups showed genetically diverse to one another. The presence of maximum genetic distance between clusters indicates that they were genetically dissimilar from each other. Similar findings previously reported [41, 42]. Exploitation of heterosis for hybrid development enabled farmers to obtain higher seed and oil yields, as well as increased uniformity. The development of sunflower hybrids set up sunflower as a major viable crop worldwide and encouraged the founding of numerous public and private breeding centers [43–45]. In recent years, public and private sector contributed to assemble huge plant genetic resources in sunflower, to identify markers for marker assisted selection (MAS) and to establish the use of new high-throughput technologies in sunflower [4, 46].

In this study, 92 SSR markers were used to discriminate the 60 hybrids with their 23 parents. Some SSR markers produced either male-specific or female specific bands that accounted for due to residual heterozygosity and even true hybrids could show the absence of any parental band [47]. Similarly, hybrids’ heterozygosity can be assessed by the presence of both parental alleles [27]. A few SSR primers have been developed and used in the sunflower for purity testing of hybrids [6]. Use of SSR primers for hybrid identification is a routine practice in many crops such as Safflower [27, 42], Rice [48], Wheat [22], Cotton [49], and Maize [50]. The reported markers in present study can be used for marker assisted introgression of the beneficial alleles. Validated molecular markers are also available which would help to develop specialty sunflower lines at lower cost, and validated markers for fertility restorer genes may help to diversify the fertility restoration sources and may improve the performance of hybrids with better grain filling under various environments [7, 49]. This study reports the applicability of SSR markers in sunflower for hybrid authentication. The characters of high polymorphism and allele size in hybrids and parents proved the potential of SSR markers in sunflower genotypic discrimination for selection of pure hybrids.

## Conclusion

The purity of a hybrid is one of the most important characteristics of good quality seed. Hybrid authentication was confirmed by the primers, which showed the polymorphic dominant loci in the parents and codominant loci midway between these parents in their related hybrids. Amplified allele size 350 bp and 330 bp for female parents L6 and L11, respectively, while, allele size 300 bp and 310 bp for male parents T1 and T2, respectively. The hybrids’ allele size was like the parents *viz*., L6×T1 (350 bp and 300 bp), L6×T2 (350 bp and 310 bp), and L11×T2 (330 bp and 310 bp). All 60 hybrids and their 23 parents were grouped into three main clusters (A, B and C) based on the results derived from genotypic data. SSR analysis performed for DNA fingerprinting of sunflower genotypes showed that 92 molecular markers gave successful results used in the fingerprinting of hybrids for their parental confirmation of sunflower in Islamic Republic of Pakistan, which would also be useful in seed purity testing and varietal discrimination at research stations and seed markets for quality and purity assurance.

## Supporting information

S1 File Supplementary tables. (DOCX)

S1 Raw images (PDF)
