# Supplementary material for: Molecular characterization and validation of sunflower (Helianthus annuus L.) hybrids through SSR markers
Source: PLoS One. 2022 May 19;17(5):e0267383. doi: 10.1371/journal.pone.0267383 (PMC9119457; doi:10.1371/journal.pone.0267383)
Supplement: S1 File — (DOCX) [file pone.0267383.s001.docx]

**Table S1: List of female parents and male parents**

| Sr. | Female parents | Origin/  Source | Sr. No. | Female parents | Origin/  Source |
| --- | --- | --- | --- | --- | --- |
| 1 | 017576 | NARC | **11** | 017596 | NARC |
| 2 | 017578 | NARC | **12** | 017598 | NARC |
| 3 | 017580 | NARC | **13** | 017600 | NARC |
| 4 | 017582 | NARC | **14** | CMS HA65 | USDA |
| 5 | 017584 | NARC | **15** | CMS HA112 | USDA |
| 6 | 017586 | NARC | **16** | CMS HA116 | USDA |
| 7 | 017588 | NARC | **17** | CMS HA207 | USDA |
| 8 | 017590 | NARC | **18** | CMS HA243 | USDA |
| 9 | 017592 | NARC | **19** | CMS HA259 | USDA |
| 10 | 017594 | NARC | **20** | CMS HA292 | USDA |
| Sr. | **Male Parents** |  |  | |  |
| 1 | 017601 | NARC |  | |  |
| 2 | 017602 | NARC |  |  |  |
| 3 | 017603 | NARC |  |  |  |

NARC, National Agriculture Research Centre, Islamabad

USDA, United States Department of Agriculture, U.S.A

**Table S2: List of cross combinations developed in this study**

| Sr. No. | Crosses | Sr. No. | Crosses |
| --- | --- | --- | --- |
| 1 | 017576 ×R-017601 | **31** | 017596× R-017602 |
| 2 | 017578 ×R-017601 | **32** | 017598× R-017602 |
| 3 | 017580× R-017601 | **33** | 017600× R-017602 |
| 4 | 017582× R-017601 | **34** | CMS HA65× R-017602 |
| 5 | 017584× R-017601 | **35** | CMS HA112× R-017602 |
| 6 | 017586× R-017601 | **36** | CMS HA116× R-017602 |
| 7 | 017588× R-017601 | **37** | CMS HA207× R-017602 |
| 8 | 017590× R-017601 | **38** | CMS HA243× R-017602 |
| 9 | 017592× R-017601 | **39** | CMS HA259× R-017602 |
| 10 | 017594× R-017601 | **40** | CMS HA292× R-017602 |
| 11 | 017596× R-017601 | **41** | 017576× R-017603 |
| 12 | 017598× R-017601 | **42** | 017578× R-017603 |
| 13 | 017600× R-017601 | **43** | 017580× R-017603 |
| 14 | CMS HA65× R017601 | **44** | 017582× R-017603 |
| 15 | CMS HA112× R017601 | **45** | 017584× R-017603 |
| 16 | CMS HA116 × R017601 | **46** | 017586× R-017603 |
| 17 | CMS HA207× R-017601 | **47** | 017588× R-017603 |
| 18 | CMS HA243× R-017601 | **48** | 017590× R-017603 |
| 19 | CMS HA259× R-017601 | **49** | 017592× R-017603 |
| 20 | CMS HA292× R-017601 | **50** | 017594× R-017603 |
| 21 | 017576× R-017602 | **51** | 017596× R-017603 |
| 22 | 017578× R-017602 | **52** | 017598× R-017603 |
| 23 | 017580× R-017602 | **53** | 017600× R-017603 |
| 24 | 017582× R-017602 | **54** | CMS HA65× R-017603 |
| 25 | 017584× R-017602 | **55** | CMS HA112× R-017603 |
| 26 | 017586× R-017602 | **56** | CMS HA116× R-017603 |
| 27 | 017588× R-017602 | **57** | CMS HA207× R-017603 |
| 28 | 017590× R-017602 | **58** | CMS HA243× R-017603 |
| 29 | 017592× R-017602 | **59** | CMS HA259× R-017603 |
| 30 | 017594× R-017602 | **60** | CMS HA292× R-017603 |

**Table S3: List of Studied SSR markers with their Forward and Reverse Sequence**

| Marker Number | **Locus** | **Forward** | **Reverse** |
| --- | --- | --- | --- |
| M1 | ORS474 | TGCACCTTTGTTTGGATCTTC | GTGCTCGGGATTGATTCTGT |
| M2 | ORS543 | CCAAGTTTCAGTTACAATCCATGA | GGTCATTAGGAGTTTGGGATCA |
| M3 | ORS959 | CCGCTAAGTATAAACCGCCTATT | CGTCCTCTTCGCATCAATCTTAT |
| M4 | ORS371 | CACACCACCAAACATCAACC | GGTGCCTTCTCTTCCTTGTG |
| M5 | ORS1035 | CAACCCAACTTCTCCTCATAACC | AGGGCTGATATTCACTTCACACA |
| M6 | ORS1145 | TCGATTACATTCATCTATGCCTAATC | CACTAGCACCCATCTCACACTTTAC |
| M7 | ORS1187 | AAAGATGTCAGTTTGTCCGTGTC | CATCCACATTTCCCTTACACTCA |
| M8 | ORS342 | TGTTCATCAGGTTTGTCTCCA | CACCAGCATAGCCATTCAAA |
| M9 | ORS1112 | CCCCATCAATCATATTTACCATGT | CGCACACTTCATCTCTCGTTACA |
| M10 | ORS1134 | ACACTGACCATACACCCAACAAC | GGCATTCTTGTCATTTCACCAT |
| M11 | ORS433 | CCGAGGTTTGATCGCTATTT | AGCGTTTGTGATTTGATTACGA |
| M12 | ORS485 | ATAAGAGCCGCCCCTGAATA | GCTAAAAGTGAACATGCTGGAA |
| M13 | ORS337 | TTGGTTCATTCATCCTTGGTC | GGGTTGGTGGTTAATTCGTC |
| M14 | ORS1285 | ATAAGTAAGACCTTGAGTCCACAGC | GATGATCTTGGGCTGATGATG |
| M15 | ORS334 | TTGGCACAATCTGAAACAAGA | AATCAAACCGAAAGCCAAACT |
| M16 | ORS366 | AACCAACTGAGCATTCTTGTGA | GCGCTAGGTTAAAGAGGACAAA |
| M17 | ORS315 | GCCGTGAATAATGGGATTGA | GATTGGGTCAGCTTGTGTGA |
| M18 | ORS481 | AGAGGATGGGCAGCTACGTC | GTCTTGACGGGCCTCCTTCT |
| M19 | ORS896 | CACAAAACAATCGCTAAAAGAACA | AATGATGATGGTCACGAAGAAGA |
| M20 | ORS533 | TGGTGGAGGTCACTATTGGA | AGGAAAGAAGGAAGCCGAGA |
| M21 | ORS516 | CCATAAACGATAGCAAATCAATTC | TAAAGGAGCCGGTGACAAGA |
| M22 | ORS1256 | GATGTTGATGTTGGTGAAGTTGC | CTCCGTCACCTTAAGCACTTGTA |
| M23 | ORS725 | TCCGACGACCAAAGAAACTT | CACAATGAAGGGAAATGGAGA |
| M24 | ORS1229 | GAAACAGAGTGAGGCAAACTTCA | CAGCGCCGTATCGTATAGAGTAA |
| M25 | ORS400 | CGAACCCGTCTGTACCGTTT | ACTTCGTTCACAAGGCACAA |
| M26 | ORS426 | CTTGACGACGGAGCTTGATG | GCGCCACTAGATAATGTGATGA |
| M27 | ORS700 | GTACCCACCACGCTTAACCA | AGTCTTCCACAGCAACGTCA |
| M28 | ORS1163 | GGACCTGACAAGCAGTTAATGAG | AAATGGATGATGATGTTGTAGTGG |
| M29 | ORS599 | TTCCCTATCACACGCCTCTC | GAAAGGAAGTAGCGGTGGTG |
| M30 | ORS453 | CCTGTGAGCTACAATACTCCCACA | GATTCTGATTAGGCGGTGGT |
| M31 | ORS762 | TGCACATGAGGGTATTCTTGTC | TCGAGGAGAGTGTGACGTTG |
| M32 | ORS780 | TGATTACAACCCTAATTCGCATAC | GATACTGGTGGGACAGATGTTG |
| M33 | ORS882 | AAACCGGCATGTAAGATATTCG | ATCGGGAGCAGAAGAAGAGTATG |
| M34 | ORS510 | CATCGCGTCCCTCTCTCTAA | CCAACCATCACAGCAATCAG |
| M35 | ORS795 | CGCTAGTTACACCGCAGATG | TGTCCACAGGTTGAAGATCG |
| M36 | ORS713 | TGAAATGTATTCACCGTCATCAG | AATCAGGGAAAGAAACGGCTA |
| M37 | ORS467 | GGGTGCATCTTGTGGAACTA | AACCGAAACATTACCCTTTCA |
| M38 | ORS749 | GGTTATCCCTTCCTTACAACTTCA | ACTCCGACGCTCAAGTCAGT |
| M39 | ORS613 | GTAAACCCTAGGTCAATTTGCAG | ATCTCCGGAAAACATTCTCG |
| M40 | ORS691 | GCATCTGAGCAACTGCGTTA | ACCGTCCTTAGCTCTTGTGAG |
| M41 | ORS990 | GGGAAACTTACTCCCTTGATGTT | GCGACACTATTATTTCACTCACTTTC |
| M42 | ORS686 | ACTAAGTGCGAGGACGAGGA | AGCACAAGCAGCAGAAGCTC |
| M43 | ORS769 | GTTTATTTATGTAGAAATGTTCTGGAA | ATGTGGTGGTAAGGGTTGTTG |
| M44 | ORS697 | TTGGGCTGTGGTTCCTTAAC | AAGAGATGGGAGTGTTGATGC |
| M45 | ORS1251 | GATGTCGGTATTGGATGTCGAT | CCAAACACCCCTAAAACTCACTC |
| M46 | ORS1040 | CTGCTGATCGTTTCTTGGATAGA | TGCTAATCCTTCTAATCAACTTCCAC |
| M47 | ORS1085 | GACCTCAAGGCATGCTAACACTC | ACTAAGTGTGTGGACGGGGAAA |
| M48 | ORS679 | CCCTCCTCCCTCTTCACTTT | CTCATCGGACAACCAGAACC |
| M49 | A11G17 | TGAACTCTTGTGTTGGCATCT | AAATGTGGATTTATGTATCTCAGTAA |
| M50 | A14M14 | AGAGGAATGAGATCGGGTTGAT | GTGGGACAACTCAGCAACGTC |
| M51 | A16A09 | TTGTTGTGGTGACTTGATTTAT | TCTCTTTAAATACACTTCCTCTCT |
| M52 | B14H20 | GACCGAATAGGTTTCAACGATAA | TTGCCTCTGCTCTCCTCTTTC |
| M53 | c0025 | CCCATTGTTGCCTAATTCAAGAT | TTGGAGTGCATAACACGTATCAG |
| M54 | c0306 | ACTGTCAACACCTCCTTCGACT | GGCTTACACTTCTCTCCATCTCAA |
| M55 | c0737 | CAACTACCCATCACTGGCAAAT | ATCACCTCCAAACATCACAAGG |
| M56 | c1111 | CGCAGATGAAACACAGGAAACT | AATATAAATGGACCTCCGCTTGA |
| M57 | c1185 | GGGCTATGGTGCGAATGTAGG | GCCTGTCAAATGTCCTTGTTGAT |
| M58 | c1628 | GCTGAAACGGGTCAATAAAGTC | TGAAGAAATGCTTCCAATCTGA |
| M59 | c1731 | GAAGTCTGCTTTGGTGTTGGTC | ATGGCTCTCTCATTTCCACTTG |
| M60 | c1779 | CATGTCCCGATCAAAGAGTTGT | CCATATCCTGGTTGTTGTGGAG |
| M61 | c2070 | GGGTAATGCAAAGTACTAAGATGTG | GCATCATCCAACAAACTAGAAGG |
| M62 | c2104 | TGGTCCCAACACTACTGATAAGG | TCAAAGGGATTCGTGAAAGTAGTT |
| M63 | c2157 | AAGAGATGCTCCACTTGTCGAA | CAGATTTAACACAGAAAGATCCATGC |
| M64 | c2293 | AACCGTAAATGAAATCGGTGTG | GAGGGCAAAGTTGGGATACTCT |
| M65 | c2443 | GAAGTTGGGAGGGTTGTTCAAG | CCTCCTGTTGGAACACCAAAT |
| M66 | c2518 | AAGCAGGTTGCATGAAGAGAAG | GTCGAAACGGGTCAGGTTGTAT |
| M67 | c3240 | TTTCGTGACCGAGAAAGGTAT | TGTGGCATACATAGAAATGATCTAA |
| M68 | c3258 | AATTAGAAGGCTATGGCACAACA | CCTTATGGCCACCACATTACAT |
| M69 | c3464 | TGTGCAGCGACGACTATAAAGA | CGTCAAACACAAATACTCCAACAA |
| M70 | c3509 | CTGCGTAGGATGATGAGGTGTC | CCAGCATCAGGTGATTCTTTCT |
| M71 | c3797 | CATTGAGGACGAGAAGCCAGT | GTTCCGTACCCTGTTTGAGCTT |
| M72 | c4098b | GGATGAGAGACCGGAGAAATA | AAAGGAAGTATTGATTTATGTATGGA |
| M73 | c4289 | CACGCCATATGAAAGCAACTCT | TCATGGATGTTCATCACCAGAC |
| M74 | E10D18 | TCAAGCAATCAGACACCACATC | TGAACACAACCAAGAAATCCAA |
| M75 | E11N15 | GTTTCGCAGCTCTAGCATTGA | CCCCCTACAAGGCAACAAAATA |
| M76 | E28P08 | GGTGGGTGTTTGATTTTGTGTG | TCTCCTCTCTCCCTTTGTCTGC |
| M77 | F7O09 | TTATGGAATGGAGTGGGAGTTG | TTGTTGGTTGATGGGATCTTGT |
| M78 | G16E04 | CTCAGGTGAACGGGATCCTTAT | CGGATTCGATTTCTTGCTTAGA |
| M79 | H9F13 | TGTTACCAGAATTGGCAAGAGC | CGCTAGAACCGAACACATCATT |
| M80 | I8D12 | CTGAGTTTCGTGTACCATTTCTATTG | ACACCAATCAGTGGGTTTCATC |
| M81 | J2J15 | AAAGGCGATTGAGTTTTTCGAT | GCAAGAATGAAAGGCCAACTTTA |
| M82 | B13L10 | CCATCCATAGACCATCATTGTG | GGGGAGCCAAAATTTCTAACA |
| M83 | B41A01 | CGCAAGTGGGTAATTTGTAGTTG | CGGCCTCTTTCTTCTTTCTTTC |
| M84 | c0233 | GTGATTGTGTGCTTTGCATGTA | CAAGGATAACATAAGTGTGTCTGG |
| M85 | c0490 | ACTTGGAAATTGGTGGATGG | TACCGTTTGCTCGTGAATTG |
| M86 | c0531 | CGGCAGTTGTTTACGATGG | TACAACACCACCAGCTCACC |
| M87 | c0578 | GATGAGGAAGTCGATGAGATG | AGGAACCCTTACACTAACAGAAA |
| M88 | c1230 | AACCAAAGATTCAAGGCAATCA | CAGACATTAGACGCGAAGCAG |
| M89 | c1357 | TTAAAGTATGCGCCGAATGTC | TTGTAGAACACCACCCTCAAC |
| M90 | c2202 | CCATTGTCTAGGGTCAAGTTCG | TTTCCACGCAATAAGCATTACTC |
| M91 | c2434 | GGCCAGTGATCGTTTATTCGT | CAAGCCCATTACATACACACCA |
| M92 | c2516 | GGAGAACGATAACGACTCTAAGGA | GACACAAGACCCTGAACAACAA |
| M93 | c3941 | TGCAAGTATGCGATCAGAGAG | GAGCAAACGCCTGGTACTTC |
| M94 | c4057 | CCATTAGCCCACTCAGATGACTT | TCAGTTGATTCCTGTTTCGTCA |
| M95 | c4230 | AAAGATCGCCGTTGAGAAGAG | TACACCTTTGACCACCGGATT |
| M96 | c4287 | GGATTGAAAGTGGTGAAATTGG | CAGCTTCCAGAACCCTTCCTC |
| M97 | ORS1041 | CTGCTGATCGTTTCTTGGATAGA | TGCTAATCCTTCTAATCAACTTCCAC |
| M98 | ORS764 | GTTTATTTATGTAGAAATGTTCTGGAA | ATGTGGTGGTAAGGGTTGTTG |
| M99 | ORS691 | GCATCTGAGCAACTGCGTTA | ACCGTCCTTAGCTCTTGTGAG |
| M100 | ORS6862 | ACTAAGTGCGAGGACGAGGA | AGCACAAGCAGCAGAAGCTC |
| M101 | ORS6130 | GTAAACCCTAGGTCAATTTGCAG | ATCTCCGGAAAACATTCTCG |
| M102 | ORS5433 | CCAAGTTTCAGTTACAATCCATGA | GGTCATTAGGAGTTTGGGATCA |
| M103 | ORS4744 | TGCACCTTTGTTTGGATCTTC | GTGCTCGGGATTGATTCTGT |
| M104 | ORS4536 | CCTGTGAGCTACAATACTCCCACA | GATTCTGATTAGGCGGTGGT |
| M105 | H9F136 | CTCAGGTGAACGGGATCCTTAT | CGGATTCGATTTCTTGCTTAGA |
| M106 | CO3064 | ACTGTCAACACCTCCTTCGACT | GGCTTACACTTCTCTCCATCTCAA |
| M107 | A14M142 | AGAGGAATGAGATCGGGTTGAT | GTGGGACAACTCAGCAACGTC |
| M108 | G16E046 | CTCAGGTGAACGGGATCCTTAT | CGGATTCGATTTCTTGCTTAGA |
| M109 | E10D18 | TCAAGCAATCAGACACCACATC | TGAACACAACCAAGAAATCCAA |
| M110 | ORS1042 | CTGCTGATCGTTTCTTGGATAGA | TGCTAATCCTTCTAATCAACTTCCAC |

**Table S4: Scorable DNA bands amplified by polymorphic SSR primers through PCR**

| **Marker code** | **Marker name** | **BP** | **PIC** | **GD** | **TNA** |
| --- | --- | --- | --- | --- | --- |
| M1 | ORS1040 | 140-180 | 0.64 | 0.72 | 5 |
| M2 | ORS769 | 240-280 | 0.68 | 0.72 | 5 |
| M3 | ORS691 | 230-270 | 0.71 | 0.72 | 5 |
| M4 | ORS6861 | 220-260 | 0.70 | 0.72 | 5 |
| M5 | ORS613 | 210-250 | 0.71 | 0.72 | 5 |
| M6 | ORS543 | 250-290 | 0.67 | 0.72 | 5 |
| M7 | ORS474 | 250-280 | 0.66 | 0.70 | 4 |
| M8 | ORS453 | 300-350 | 0.76 | 0.72 | 6 |
| M9 | H9F13 | 320-370 | 0.74 | 0.72 | 6 |
| M10 | CO306 | 300-350 | 0.74 | 0.72 | 6 |
| M11 | A14M14 | 360-400 | 0.72 | 0.70 | 5 |
| M12 | G16E04 | 350-390 | 0.69 | 0.72 | 5 |
| M13 | E10D18 | 310-330 | 0.67 | 0.68 | 4 |
| M14 | c3797 | 320-350 | 0.65 | 0.72 | 4 |
| M15 | C3464 | 240-280 | 0.70 | 0.72 | 5 |
| M16 | C3258 | 180-210 | 0.70 | 0.69 | 5 |
| M17 | C2516 | 220-250 | 0.65 | 0.72 | 4 |
| M18 | C2293 | 300-340 | 0.69 | 0.72 | 5 |
| M19 | B41A01 | 330-360 | 0.67 | 0.72 | 4 |
| M20 | C1779 | 350-390 | 0.70 | 0.72 | 5 |
| M21 | C1628 | 340-390 | 0.73 | 0.71 | 6 |
| M22 | C2443 | 230-270 | 0.70 | 0.72 | 5 |

PIC (polymorphic information contents), BP (Base pair Position) GD (genetic diversity of markers/ heterozygosity) , TNA (Total no of alleles)
