# Supplementary material for: Molecular characterization and validation of sunflower (Helianthus annuus L.) hybrids through SSR markers
Source: PLoS One. 2022 May 19;17(5):e0267383. doi: 10.1371/journal.pone.0267383 (PMC9119457; doi:10.1371/journal.pone.0267383)
Supplement: S1 Raw images — (PDF) [file pone.0267383.s002.pdf]

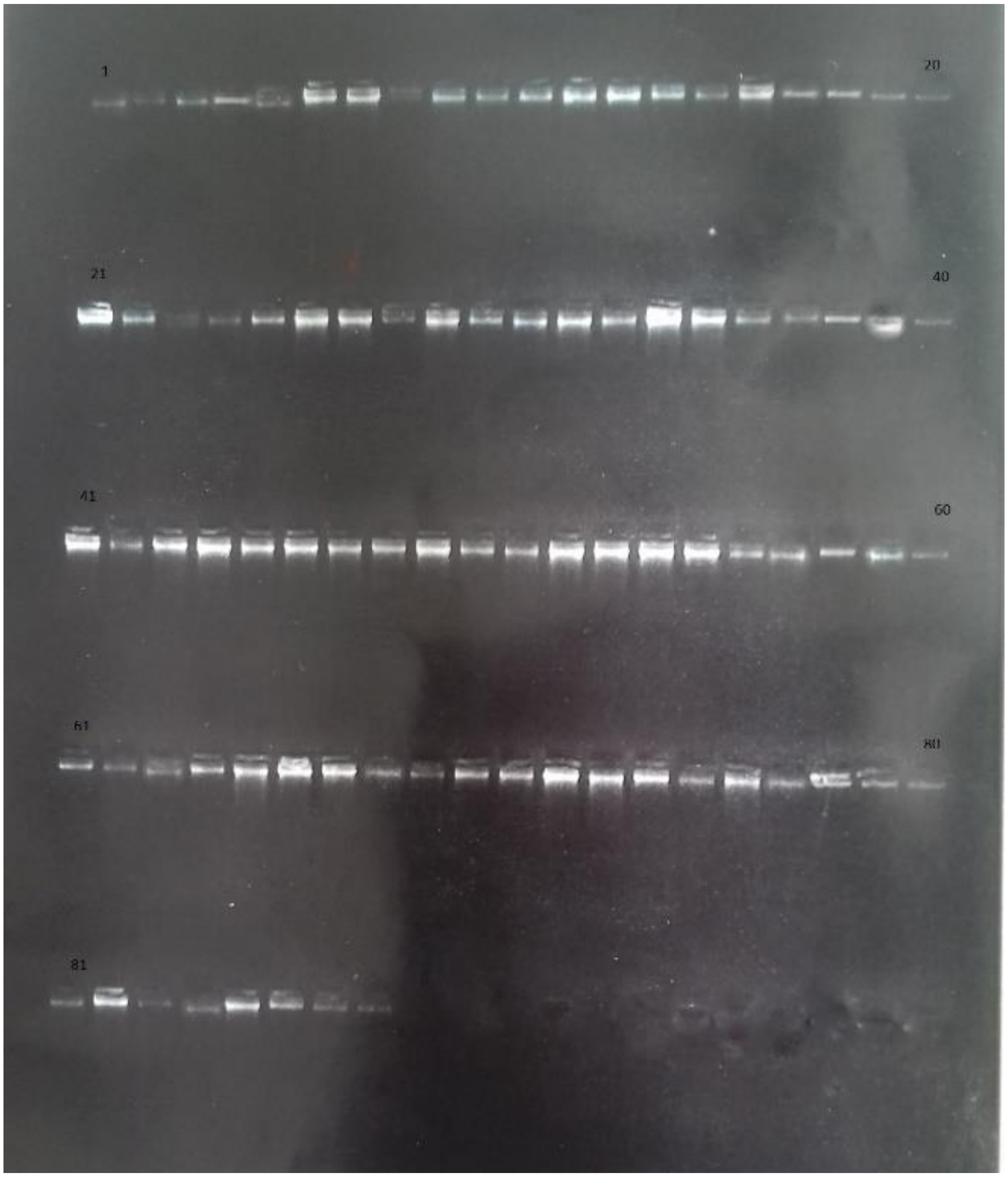

Fig.S1: Genomic DNA of all the 83 (Parents and their Crosses) genotypes visualized on gel electrophoresis

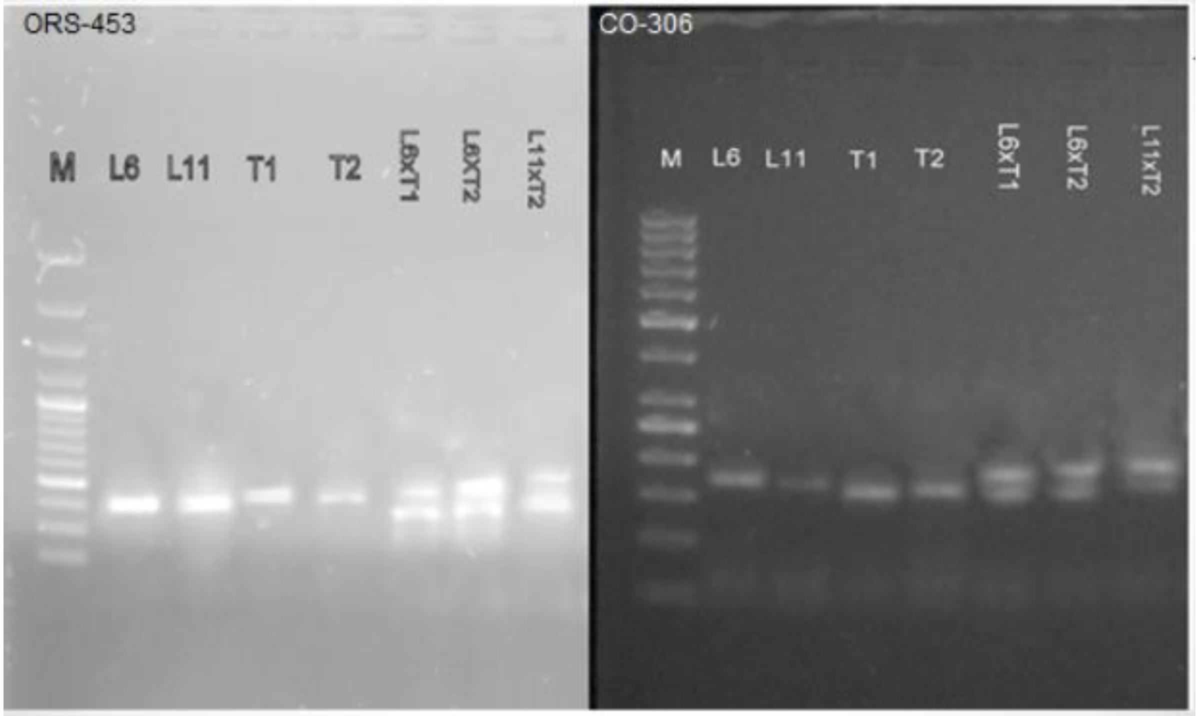

Fig. S2. Gel picture showing hybrid authentication with their respective parents using ORS453 and CO306.
